# Types of diabetes are not limited to age groups: type 1 diabetes in adults and type 2 diabetes in children and adolescents

**DOI:** 10.25646/5987

**Published:** 2019-06-27

**Authors:** Joachim Rosenbauer, Andreas Neu, Ulrike Rothe, Jochen Seufert, Reinhard W. Holl

**Affiliations:** 1 German Diabetes Center, Leibniz Center for Diabetes Research at Heinrich-Heine-Universität Düsseldorf, Institute for Biometrics and Epidemiology; 2 German Center for Diabetes Research (DZD), München-Neuherberg; 3 University Children’s Hospital Tübingen; 4 Technische Universität Dresden, Faculty of Medicine Carl Gustav Carus, Health Sciences/Public Health; 5 Division of Endocrinology and Diabetology, Department of Medicine II, Medical Center – University of Freiburg, Faculty of Medicine, University of Freiburg; 6 Ulm University, Institute of Epidemiology and Medical Biometry, ZIBMT

**Keywords:** TYPE 1 DIABETES, TYPE 2 DIABETES, INCIDENCE, PREVALENCE, DIABETES SURVEILLANCE

## Abstract

Based on data from the national diabetes registry DPV (Diabetes patient documentation), the diabetes registry of North Rhine-Westphalia and surveys conducted at hospitals and practices in Baden-Württemberg and Saxony, this study estimates the incidence and prevalence of type 1 diabetes in over-18-year-old adults and type 2 diabetes in 11- to 18-year-old children and adolescents.

The national incidence of type 1 diabetes in adults was 6.1 per 100,000 person-years from 2014 to 2016, with slightly lower figures for women compared to men. Annually, around 4,150 adults develop type 1 diabetes. In 2016, the prevalence of type 1 diabetes was estimated at 493 per 100,000 persons and was lower in women at 445 per 100,000 people than in men at 544 per 100,000. Based on this data, there were around 341,000 adults with type 1 diabetes in 2016.

For 11- to 18-year-old children and adolescents, the national incidence of type 2 diabetes was 2.8 per 100,000 person-years between 2014 and 2016 and higher for girls than for boys. Annually, around 175 adolescents in this age group develop type 2 diabetes. The incidence estimates for Saxony were higher (4.3 per 100,000 person-years). The prevalence of type 2 diabetes between 2014 and 2016 for 11- to 18-year-old children and adolescents was estimated between 12 and 18 cases per 100,000 persons. During this period, there were about 950 children and adolescents of this age group with type 2 diabetes in Germany.

## 1. Introduction

Diabetes mellitus means a chronic increase of glucose levels in the body. From a health policy perspective, two forms of diabetes in particular play an important role: immune-mediated type 1 diabetes, which generally leads to absolute insulin deficiency, and type 2 diabetes, in which both insulin resistance as well as reduced insulin secretion play a role. The disease reduces both life expectancy and quality of life, mainly due to chronic damage to small and large blood vessels. Data on the incidence and prevalence of diabetes, therefore, provide important information to build and further develop a diabetes care infrastructure.

Three regularly updated regional paediatric diabetes incidence registries from Baden-Württemberg, North Rhine-Westphalia (NRW) and Saxony, as well as the national DPV registry, provide sound data on the incidence of type 1 diabetes in under-15-year-old children and adolescents. These data sources also indicate the long-term trends for the incidence of type 1 diabetes in this age group [1-3]. In addition, the data from these three regional incidence registries feed into the European surveillance of type 1 diabetes within the framework of the EURODIAB (Epidemiology and Prevention of Diabetes) study group [4-6]. The project described here aims to make this data available for national level diabetes surveillance too.

However, only limited information is available on the incidence of type 1 diabetes among adults. Based on the data from statutory health insurances, national prevalences across all age groups in 2009 and 2010 were estimated at 300 cases per 100,000 persons [7]. Accordingly, there were around 256,000 persons with type 1 diabetes in Germany in 2009 and 252,000 in 2010. Germany-wide claims data between 2009 and 2015 estimate similar prevalences, as well as a downward trend from 330 to 280 cases per 100,000 persons [8]. Projected for the German population, around 230,000 persons, accordingly, had type 1 diabetes in 2015. Reports from the disease management programs (DMP) of statutory health insurances estimated a significantly higher figure of around 311,000 persons with type 1 diabetes across all age groups for 2014 [9]. According to the DMP quality report for type 1 diabetes in Westfalen-Lippe, there were 22,807 DMP registered patients across all age groups in 2015 [10]. This corresponds to a prevalence of 314 per 100,000 persons among those covered by statutory health insurance. Taking into account the estimated completeness of DMP in Westfalen-Lippe (78.4% to 89.4%), this would result in a prevalence of 351 to 400 per 100,000 persons [10].

Based on 2009 and 2010 statutory health insurance data, national age-specific incidences of type 1 diabetes were estimated for the first time for the age group up to 55 years of age [11]. The incidence for 15- to 55-year olds was 7.1 and 6.1, respectively, per 100,000 person-years.

A better data situation is given for type 2 diabetes in adulthood. A number of national and representative regional studies have analysed the prevalence and incidence of known and unknown diabetes. Various publications present the results of these studies [3, 12, 13]. In particular, there are estimates based on the data from statutory health insurances and claims data from statutory health insurance-authorised physicians [7, 8].

For type 2 diabetes in adolescents, however, there is only insufficient data available. A study conducted in Baden-Württemberg between the years 2004 and 2005 reported the prevalence of known type 2 diabetes in 0- to 20-year-old children and adolescents as 2.3 per 100,000 persons [14]. For North Rhine-Westphalia, the prevalence among 5- to 19-year-old children and adolescents in 2010 was estimated at 5.8 per 100,000 persons. According to the study, 600 to 800 5- to 19-year-old children and adolescents had a known type 2 diabetes in 2010 [15]. According to data from the nationwide DPV register, in the last 10 years about 5% to 6% of children and adolescents aged 11 to 18 years newly diagnosed with diabetes have type 2 diabetes [16]. Based on 2009 and 2010 statutory health insurance data, the national prevalence for under-20-year-old girls and boys was estimated to be much higher at 30 and 40 cases, respectively, per 100,000 persons [7]. On the basis of claims data from all over Germany, the prevalence in 2009 and 2015 for 0- to 19-year olds was estimated as 66 and 41 cases per 100,000 persons, respectively [3]. However, it is a well-known fact that claims data leads to an overestimation of the prevalence, also because other forms of diabetes, when no insulin therapy is prescribed, are erroneously categorised as type 2 diabetes.

The diabetes registry of North Rhine-Westphalia provides estimates for the incidence of type 2 diabetes in children and adolescents. Recently, an average incidence of 1.3 per 100,000 person-years was estimated for the period 2002 to 2014 for children and adolescents aged 5 to 19 years, whereby the incidence between 2011 and 2014 was 1.6 per 100,000 person-years. Accordingly, 130 to 160 persons in the 5- to 19-year-old age group developed type 2 diabetes annually. Again, based on Germany-wide claims data for the 2012 to 2014 period, a 15-fold higher incidence of 20 and 30 cases per 100,000 person-years was estimated for girls and boys under the age of 20, respectively. [8].

So far, a continuous provision of estimates based on a standardised methodology to account for the incidence and prevalence of type 1 diabetes across the entire adult age spectrum, as well as of type 2 diabetes in adolescents, has not been established in Germany, although such data would be relevant to diabetes surveillance and the further development of the infrastructure of health care provision. Precisely, because many diabetes prevention approaches target the group of young, at risk people, surveillance would need to reliably and promptly show changes in the incidence in this age group. The objectives of this project in the context of diabetes surveillance at the Robert Koch Institute (RKI) were therefore to network with existing structures and develop supplementary structures for the continuous monitoring of the regional and the national incidence and prevalence in this group of patients.

## 2. Methodology

### 2.1. Definition of type 1 and type 2 diabetes

Our analysis uses the term type 1 diabetes only in the sense of ‘classical’ clinically diagnosed type 1 diabetes. The practice guidelines of the German Diabetes Association also classify patients with latent autoimmune diabetes in adults (LADA) as type 1 diabetes, i.e. a form of immune-mediated diabetes, which in most cases leads to complete insulin insufficiency in patients shortly after the onset of diabetes [17]. Diagnosing this form of diabetes requires complex laboratory examinations. However, an examination of all newly diagnosed adult diabetes patients for beta-cell antibodies (indicating the immune reaction against insulin-producing cells in the pancreas) and for C-peptide (reflecting the remaining insulin secretion by islet cells), is currently not diagnostic standard. This lack of standardisation means that results are only comparable to a limited extent, we therefore have not categorised LADA as type 1 diabetes. The diagnosis of type 2 diabetes is based on the German Diabetes Association’s current guidelines [18].

### 2.2 Data sources

Table 1 provides an overview of the data sources used. To estimate the incidence and prevalence of type 1 diabetes in adults (≥ 18 years) in North Rhine-Westphalia and nationwide, the population-based diabetes registry of North Rhine-Westphalia [1] and the national DPV registry [19] were used as data sources.

The North Rhine-Westphalian registry has collected data for 0- to 14-year-old children and adolescents since 1996. It has recorded newly diagnosed cases of type 1 and type 2 diabetes in the 0- to 34-year-old age group since 2002 based on three data sources: the clinic-based German Paediatric Surveillance Unit (ESPED), annual surveys of medical specialists working in private practices, paediatricians, general practitioners, as well as the national DPV registry.

The DPV initiative was started throughout Germany in 1995 and is a computer-aided longitudinal collection of data on treatment of diabetes patients and outcomes. For the diabetes surveillance at the RKI, only the data from German centres (420 institutions: 181 internal medicine and 239 paediatric centres) was used. Data for older age groups is presumably far less complete, because DPV data collection only began in the mid 1990s and patients aged over 35 years in 2016 would have developed the disease well before 1990.

The incidence and prevalence of type 2 diabetes in 11- to 18-year-old children and adolescents in North Rhine-Westphalia and at the national level was estimated by using the population-based diabetes registry of North Rhine-Westphalia and the nationwide DPV registry. In addition, data on incidence was collected in Saxony (11- to 18-year-olds) and data on prevalence in Baden-Württemberg (under 20-year-olds).

The data source in Saxony was a survey (by post, telephone or e-mail) on the number of new cases of type 2 diabetes in children and adolescents (11 to 18 years) in 2016 among the clinics participating in Saxony’s diabetes registry (all 31 paediatric clinics) and specialised diabetological practices [20, 21].

The data source in Baden-Württemberg was a survey (by telephone, e-mail or fax) on the number of children and adolescents with type 2 diabetes (under 20 years of age) treated in 2016 among the participating clinics of the DIARY registry (Diabetes Incidence Registry in Baden-Württemberg, 31 paediatric clinics and one diabetes centre) [22, 23], the 242 members of the German association of diabetologists (BVND) in Baden-Württemberg as well as the 266 members of the working group diabetes in Baden-Württemberg (ADBW). An initial written request was made in July 2017, a second request in September 2017. According to an earlier analysis of the prevalence of type 2 diabetes and the available care in Baden-Württemberg, around 50% of type 2 diabetes patients aged under 20 years of age are treated in paediatric clinics, 39% by office-based diabetologists, 7% by diabetologists in clinics, 2% in clinics and 2% in practices without diabetologists. The current survey of paediatric clinics and office-based diabetologists therefore covers around 90% of all cases.

The population data necessary to estimate incidence and prevalence was obtained from the Federal Statistical Office. Unless otherwise stated, the population data result from an update of the population level for 2015 on the basis of the 2011 census [24], since the population data for North Rhine-Westphalia (and Baden-Württemberg) were not yet available for 2016 at the time of the analysis. This also applies to the calculations for person years 2014 to 2016, which are based on 2014 and two times on 2015.

### 2.3 Statistical methods

The recording of new cases of diabetes by the registry of North Rhine-Westphalia, which is based on three data sources, allows estimating the completeness of the data collected by applying capture-recapture methods. Thereby, the proportion of patients who appear in more than one data source allows an estimate of the number of patients who were not recorded. Subsequently, the figures can then be adjusted. To estimate the completeness of the data collected, a suitable statistical model was adapted to the data (log-linear model that accounts for overdispersion) [25, 26]. After applying a standard statistical criterion (AICC [27]), the model was chosen that best describes the available registry data. Both the completeness of the registry in general and, in addition, of the DPV database was estimated.

Estimates regarding the completeness of the North Rhine-Westphalia registry were used to calculate estimates corrected for underreporting for the incidence and prevalence in North Rhine-Westphalia from the data observed in the North Rhine-Westphalia registry. Estimates for the completeness of the DPV data basis for North Rhine-Westphalia were used to calculate ascertainment corrected estimates of incidence and prevalence for Germany in national DPV data. Based on the North Rhine-Westphalia and DPV registries, completeness of ascertainment could only be estimated for the 18- to 34-year-old age group; it was therefore necessary to make a plausible assumption for the completeness of the DPV registry for older age groups. The presumed completeness of ascertainment for older age groups was varied in terms of a sensitivity analysis. Incidence and prevalence with 95% confidence intervals (95%-CI, range in which the true value lies with 95% certainty) were estimated applying a Poisson distribution [28, 29]. A detailed description of the methodology can be found in [25-29].

## 3. Results

### 3.1 Incidence of type 1 diabetes

#### Incidence of type 1 diabetes in 18- to 34-year-olds in North Rhine-Westphalia between 2014 and 2016

Between 2014 and 2016, the Rhine-Westphalian diabetes registry recorded 391 newly diagnosed type 1 diabetes patients in the 18- to 34-year-old age group. This is an incidence of 3.6 per 100,000 person-years. The registry captured an estimated 29.5% of the population; adjusted for undereporting, the incidence was therefore estimated at 12.0 per 100,000 person-years (Table 2). Correspondingly, the absolute figure for new cases between 2014 and 2016 was 1,326, i.e. an average of 442 new cases per year. Adjusted for undereporting, the incidence for women was only about half that for men (7.9 vs. 16.1 per 100,000 person-years) and the incidence was slightly higher for 18- to 24-year-olds than for 25- to 34-year-olds (12.9 vs. 11.7 per 100,000 person-years). Table 2 also shows the incidence for registered cases by age and gender.

#### Incidence of type 1 diabetes in adults over 18 years of age in Germany between 2014 and 2016

Between 2014 and 2016, 2,144 patients at least 18 years of age were registered in the DPV diabetes registry with newly diagnosed type 1 diabetes. Against 205,986,915 person-years [31], this is an incidence of 1.0 per 100,000 person-years (Table 2). The ascertainment rate of DPV for the 18- to 34-year-old age group was estimated at 17.2% based on the North Rhine-Westphalia registry. Assuming that DPV has the same capture rate in older age groups too, the incidence in the age group 18 years and older adjusted for undereporting was estimated as 6.1 per 100,000 person-years. Accordingly, there was an absolute number of 12,495 new cases between 2014 and 2016, i.e. an average of 4,165 new cases per year. Assuming a completeness of DPV in the age group over 35 years of 22.2% or 12.2% (17.2±5%), this gives an adjusted incidence estimate of 5.3 (95% CI 5.2-5.4) or 7.4 (95% CI 7.3-7.5) per 100,000 person-years. The absolute number of new cases was then estimated as 3,653 and 5,082.

The incidence adjusted for undereporting of type 1 diabetes was slightly lower for women compared to men (5.4 vs. 5.8 per 100,000 person-years) and decreased continuously with age, from 13.5 per 100,000 person-years in 18- to 24-year-olds to 1.4 per 100,000 person-years in the group aged at least 75. Table 2 moreover shows the incidence for registered cases by age and gender as well as corresponding estimates of ascertainemnet rates and incidences.

#### Incidence and number of new cases of type 1 diabetes among children and adolescents aged 0 to 17 years in Germany

To provide a fuller picture, we include previously published figures on paediatric type 1 diabetes patients. According to current estimates from North Rhine-Westphalia, the incidence for type 1 diabetes in the 0- to 17-year-old age group is 23.6 per 100,000 person-years, so that for Germany we can assume a figure of 3,100 new cases of type 1 diabetes annually [30].

#### Total number of new cases of type 1 diabetes for Germany (all age groups)

Based on these estimates, annually, around 7,265 persons newly develop type 1 diabetes in Germany.

### 3.2 Prevalence of type 1 diabetes

#### Prevalence of type 1 diabetes in 18- to 34-year-olds in North Rhine-Westphalia between 2014 and 2016

**Figure 1:**
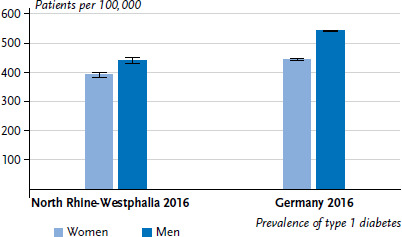
Underreporting-adjusted prevalence of type 1 diabetes according to gender in the age groups 18 to 34 years (North Rhine-Westphalia) and 18 years and older (projected for Germany) Source: Diabetes registry of North Rhine-Westphalia, Diabetes patient documentation (DPV registry) [1, 19]

In 2016, 11,285 patients with type 1 diabetes aged 18 to 34 years were registered in the North Rhine-Westphalian diabetes registry. Against a total population of 3,713,823 [31], the prevalence was 303.8 per 100,000 persons (Table 2). The estimated coverage rate was 72.5%, and the underreporting-adjusted prevalence was therefore estimated at 418.8 per 100,000 persons. Accordingly, in 2016, around 15,554 adults in the 18- to 34-year-old age group had type 1 diabetes in North Rhine-Westphalia. Adjusted for underreporting, the prevalence was 426.4 per 100,000 persons in 2014 and 413.4 per 100,000 persons in 2015. In 2016, the underreporting-adjusted prevalence for women was around 12% to 13% lower than for men (392.1 vs. 442.5 per 100,000 persons, Figure 1) and the adjusted prevalence was significantly higher for 25- to 34-year-olds compared to 18- to 24-year-olds (465.0 vs. 388.3 per 100,000 persons). Furthermore, Table 2 shows the prevalence for registered cases by age and gender as well as the corresponding coverage rates and prevalence estimates.

#### Prevalence of type 1 diabetes in adults over 18 years of age in Germany between 2014 and 2016

In 2016, 83,215 patients with type 1 diabetes aged 18 years or older were registered in the DPV diabetes registry. Against a population of 69,051,391 (as at 31.12.2016) [31], the prevalence was 120.5 per 100,000 persons (Table 2). The coverage rate of DPV in the 18- to 24 and 25- to 34-year-old age group was estimated as 79.1% and 42.6%, respectively. Assuming a DPV coverage rate of 15% in the higher age groups, a prevalence of 493.3 per 100,000 persons was estimated for the age group of 18 years or older in 2016. For 2015 and 2014, the underreporting-adjusted prevalence was 481.1 and 476.4 per 100,000 persons, respectively. Correspondingly, in 2016, 340,664 adults had type 1 diabetes in Germany. Assuming a DPV coverage rate of the age group over 35 years of 20% and 10%, respectively, an adjusted prevalence estimate of 399.3 (95% CI 397.8-400.8) and 681.5 (95% CI 679.6-683.5) per 100,000 persons resulted for 2016. The absolute number of adults with type 1 diabetes was then estimated to be 275,701 and 470,591, respectively, in 2016.

The underreporting-adjusted prevalence in 2016 was around 18% lower for women than for men (445.0 vs. 543.5 per 100,000 persons, Figure 1). Moreover, in 2016, the underreporting-adjusted prevalence rose from 425.0 per 100,000 persons in the 18- to 24-year-old age group to 683.6 per 100,000 persons in the 35- to 44-year-old age group and then decreased with age continuously to 419.3 per 100,000 persons aged 75 and over (Table 2).

#### Prevalence and number of children and adolescents aged 0 to 17 years with type 1 diabetes in Germany

According to current estimates from North Rhine-Westphalia, the prevalence of type 1 diabetes in the 0- to 17-year-old age group was 240 per 100,000 persons, which means that around 32,000 children and adolescents in Germany had type 1 diabetes [32].

#### Total number of persons with type 1 diabetes in Germany

Based on these estimates, the total number of type 1 diabetes patients across all age groups in Germany is currently almost 373,000.

### 3.3 Incidence of type 2 diabetes in children and adolescents

#### Incidence of type 2 diabetes in children and adolescents aged 11 to 18 years in North Rhine-Westphalia between 2014 and 2016

Between 2014 and 2016, 105 patients with newly diagnosed type 2 diabetes aged 11 to 18 years were registered in the North Rhine-Westphalian diabetes registry. Against a total of 4,308,426 person-years, the incidence was 2.4 per 100,000 person-years (Table 3). The registry coverage rate was 71.9%, and the estimated underreporting-adjusted incidence, correspondingly, 3.4 per 100,000 person-years. Accordingly, there were an absolute number of 146 new cases between 2014 and 2016, which is an average annual incidence of 49 cases. For girls, the underreporting-adjusted incidence rate was over 60% higher than for boys (4.2 vs. 2.6 per 100,000 person-years). No significant difference was found between the 11- to 14-year-old and the 15- to 18 year-old age group (3.5 vs. 3.3 per 100,000 person-years). Table 3 also shows the incidence for registered cases by age and gender, as well as the corresponding estimates of coverage rates and incidences.

#### Incidence of type 2 diabetes in children and adolescents aged 11 to 18 years in Saxony in 2016

The 2017 interview surveys in Saxony achieved response rates of 100% among paediatric hospitals (n=31) and 88.1% among diabetological practices (n=119). In 2016, ten new cases of type 2 diabetes were registered, three cases in paediatric clinics and seven in diabetological practices. Against a total of 252,919 person-years, this was an incidence of 4.0 per 100,000 person-years (Table 3). Adjusted for the response rate, the incidence was 4.3 per 100,000 person-years. For 2014 to 2015, previous surveys estimated an incidence – adjusted for the estimated coverage rate – of 5.1 per 100,000 person-years.

#### Incidence of type 2 diabetes in children and adolescents aged 11 to 18 years in Germany between 2014 and 2016

Between 2014 and 2016, 273 patients with newly diagnosed type 2 diabetes aged 11 to 18 years were registered in the DPV diabetes registry. With a total of 18,774,057 person-years [31], this resulted in an incidence of 1.5 per 100,000 person-years (Table 3). The DPV coverage rate was 51.6%, so that a underreporting-adjusted incidence of 2.8 per 100,000 person-years was estimated. Accordingly, there was an absolute number of 529 new cases between 2014 and 2016, i.e. an average of 176 new cases per year. Adjusted for underreporting, the incidence for girls was 50% higher than for boys (3.4 vs. 2.3 per 100,000 person-years). Adjusted for underreporting, the incidence in the 15- to 18-year-old age group was 2.5 times higher than in the 11- to 14-year-old age group (4.0 vs. 1.6 per 100,000 person-years). Table 3 moreover shows the incidence for recorded cases by age and gender, as well as corresponding estimates of coverage rates and incidences.

### 3.4 Prevalence of type 2 diabetes in children and adolescents

#### Prevalence of type 2 diabetes in children and adolescents aged 11 to 18 years in North Rhine-Westphalia between 2014 and 2016

In 2016, 150 patients with type 2 diabetes aged 11 to 18 years were registered in the diabetes registry of North Rhine-Westphalia. For a population of 1,437,776 adolescents of this age group [31], this is a prevalence of 10.4 per 100,000 persons (Table 3). The estimated coverage rate was 77.3%, and the underreporting-adjusted prevalence was therefore estimated at 13.5 per 100,000 persons. For 2015, the underreporting-adjusted prevalence was 15.2, and for 2014 14.7 per 100,000 persons. Accordingly, in 2016, 194 11- to 18-year-old children and adolescents had diagnosed type 2 diabetes, 218 in 2015 and 211 in 2014. In 2016, the underreporting-adjusted prevalence was 70% higher for girls than for boys (17.2 vs. 10.1 per 100,000 persons) and around three times higher for 15- to 18-year-olds compared to 11- to 14-year-olds. Table 3 also shows the prevalence for recorded cases by age and gender as well as the corresponding coverage rates and prevalence estimates.

#### Prevalence of type 2 diabetes in children and adolescents under 20 years of age in Baden-Württemberg

In the 2017 survey in Baden-Württemberg, the response rate for the paediatric DIARY network (31 paediatric clinics and 1 diabetes centre) was 78.1% (25/32) and for diabetologists caring for adults 21.4% (57/266) (BVND 14.5% (35/242), ADBW 8.3% (22/266)).

In 2016, a total of 80 patients with type 2 diabetes under the age of 20 years were recorded in Baden-Württemberg (Table 3), 34 patients in the DIARY network, i.e. in paediatric care, 26 BVND patients and 20 patients in the regional ADBW association. For a population of 2,097,929 children and adolescents [31], this is a prevalence of 3.8 per 100,000 persons. Taking account of the estimated 90% coverage rate provides a prevalence of 4.2 per 100,000 persons. A previous estimate had reported a lower prevalence of 2.4 (95% CI 1.8-3.1) and a lower adjusted prevalence of 2.7 (95% CI 2.0-3.5) per 100,000 persons.

#### Prevalence of type 2 diabetes in children and adolescents aged 11 to 18 years in Germany between 2014 and 2016

**Figure 2:**
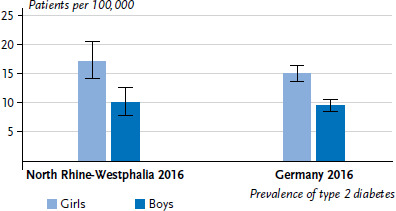
Underreporting-adjusted prevalence of type 2 diabetes according to gender in the age group 11 to 18 years Source: Diabetes registry of North Rhine-Westphalia, Diabetes patient documentation (DPV registry) [1, 19]

In 2016, 445 patients with type 2 diabetes aged 11 to 18 years were registered in the DPV diabetes registry. Against a population of 6,237,040 children and adolescents (as at 31.12.2016) [31], this is a prevalence of 7.1 per 100,000 (Table 3). Coverage rate was an estimated 58.7%, giving an estimated underreporting-adjusted prevalence of 12.2 per 100,000 persons. The underreporting-adjusted prevalence was 15.4 for 2015 and 18.2 per 100,000 persons for 2014. Accordingly, in 2016, 758 11- to 18-year-old children and adolescents had type 2 diabetes. In 2014 and 2015, it was 1,136 and 968 children and adolescents, respectively, i.e. an average of around 950 children and adolescents in the years 2014 to 2016. In 2016, the underreporting-adjusted prevalence was nearly twice as high for girls compared to boys (15.1 vs. 9.6 per 100,000 persons, Figure 2) and around 2.5 times higher for 15- to 18-year-olds compared to 11- to 14-year-olds. Table 3 also shows the prevalence for recorded cases by age and gender as well as the corresponding coverage rates and prevalence estimates.

## 4. Discussion

### 4.1 Results

#### Type 1 diabetes

For the first time, current estimates for the incidence of type 1 diabetes across all age groups for adults aged over 18 are provided. For young adults aged 18 to 34 years, the incidence estimates from North Rhine-Westphalia fit well to national incidence figures (12.0 vs. 11.8 per 100,000 person-years). For the group of all adults (aged over 18) a lower incidence of an estimated 6.1 per 100,000 person-years was found, which reflects the decreasing incidence of type 1 diabetes with age. Statutory health insurance data provides a similar incidence, yet only for the limited age ranges 15 to 55 (7.1 per 100,000 person-years) and 20 to 55 (6.1 per 100,000 person-years) [11]. As the incidence of type 1 diabetes decreases with age, the incidence estimates for all adults based on statutory health insurance data should be lower.

According to the current prevalence estimate (493 per 100,000 persons), about 341,000 adults at the age of at least 18 years are affected by type 1 diabetes at the national level. This estimate is higher than previous ones based on statutory health insurance data. According to the reports of the structured treatment programs run by statutory health insurances (DMP programs), there were around 279,000 type 1 diabetes patients over 18 years of age in Germany in 2014 [9]. Prevalence estimates based on statutory health insurance data estimated that there were 229,000 adults aged over 20 with type 1 diabetes in Germany in 2009 and 224,000 in 2010. Prevalences calculated on the basis of claims data yielded 262,000 type 1 diabetes patients in 2009 and 230,000 in 2015 [3]. However, because they fail to account for the privately insured – around 10% of the population – incidence and prevalence estimates based on statutory health insurance data most likely underestimate the true number of cases.

Combined with estimates for the 0- to 17-year-old age group, there are around 7,250 new cases of type 1 diabetes annually in Germany and there is a total of about 373,000 type 1 diabetes patients.

#### Type 2 diabetes

At the turn of the millennium, reports on a significant increase of cases of type 2 diabetes at adolescent age began to multiply. Such reports came mainly from North America and Asia, and were based on an increase in the obesity prevalence in this age group [33, 34]. This was contrasted by European surveys, which pointed at a significantly lower prevalence of type 2 diabetes in adolescents [14, 35-37].

Between 2004 and 2006, the first comprehensive cross-sectional study of type 2 diabetes among children and adolescents was conducted in Baden-Württemberg [14]. With its population of 10.7 million, of which 22.7% are under 20 years of age, Baden-Württemberg is the third largest federal state. With the DIARY network (Diabetes Incidence Registry), Baden-Württemberg has a federal state wide comprehensive surveying structure that binds in all paediatric clinics in the state, as well as one diabetes centre. The first cross-sectional survey was conducted within the framework of this network and with the participation of internal medicine clinics and diabetological practices. In 2016, ten years later, a new cross-sectional survey was conducted as a sub-project with the national diabetes surveillance at the RKI. Both DIARY clinics, all ADBW (working group diabetes in Baden-Württemberg) members, as well as the BVND (German association of diabetologists) in Baden-Württemberg took part in the survey.

The first prevalence survey in Baden-Württemberg was conducted between 2004 and 2006 and indicated a prevalence of 2.4 per 100,000 persons (95% CI 1.7-3.1) for the age group under 20 years. For 2015, the prevalence was 2.3 per 100,000 persons (95% CI 1.7-2.9). During a ten year period, the prevalence of type 2 diabetes in adolescents therefore remained stable [14]. An update within the context of this sub-project of the national diabetes surveillance showed a prevalence of 2.4 (adjusted for undereporting 2.7) for 2015 for 100,000 persons, yet a significantly higher prevalence of 3.8 (adjusted for underreporting 4.2) per 100,000 persons for 2016.

Point estimates for the incidence of type 2 diabetes vary significantly for North Rhine-Westphalia, Saxony and Germany overall, yet taking into account the uncertainty of estimates relativises these differences. All current estimates are higher than the average incidence in North Rhine-Westphalia between 2002 and 2014, a fact which indicates an increase in the rate of new cases. The incidence between 2012 and 2014 for under-20-year-olds estimated on the basis of claims data is many times higher [8]. Presumably this is due to the differences in forms of data collection and different case definitions. The observed changes to the incidence in Saxony are probably related to random fluctuations in incidence of this ultimately rare disease, as well as the potential factor of differences in the quality of data collection. Due to the great uncertainty of estimates (broad confidence intervals), evidence for incidence changes is lacking and results from longer periods of observation are to be seen.

Point estimates for the prevalence of type 2 diabetes in North Rhine-Westphalia, Baden-Württemberg and Germany overall likewise show substantial discrepancies. Particularly notable is the low prevalence in Baden-Württemberg (2015/2016: 2.4/3.8 and adjusted for underreporting 2.7/4.2 per 100,000 persons) compared to North Rhine-Westphalia and nationwide estimates (12 – 18 per 100,000 persons). Based on statutory health insurance data, the estimates for the prevalence for under-20-year-olds were many times higher. For 2009 to 2010, the prevalence was estimated as 30 to 40 per 100,000 persons [7]. Age-specific estimates reported in [8] provided an estimated prevalence of 66 or 41 per 100,000 persons for 2009 and 2015, respectively. The great discrepancy with regard to other surveys is presumably linked to differences in surveying methods and a different classification of type 2 diabetes.

All data sources point to a higher number of female adolescent type 2 diabetes cases. Higher body fat and a lower insulin sensitivity, as well as lower levels of physical activity among obese female children and adolescents could play a causal role here.

The fact that the frequency of type 2 diabetes in this age group in central Europe is far lower than for example in North America can be explained by the ethnic composition of the population. People of Afro-American and Hispanic-American background, from Asia and the Pacific Islands, as well as Native Americans have a significantly higher type 2 diabetes risk and are not an important proportion of the European population [38]. The prevalence rates found in Baden-Württemberg are comparable to those found in neighbouring countries such as Sweden [36], the UK [35] or Austria [37]. Compared to North America and Asia, type 2 diabetes among children and adolescents therefore remains a (still) relatively rare disease in Germany [39].

While the observed regional differences in the incidence and prevalence of type 2 diabetes among children and adolescents may point to actual regional differences, they could also be related to differences in surveying methods and coverage rates. Regional differences in the disease rate of type 2 diabetes among adults, in particular a North-South and an East-West gradient have been described by the DIAB-CORE (Diabetes-Collaborative Research of Epidemiologic Studies) project of the diabetes competency network and secondary analyses of statutory health insurance data [8, 40, 41]. Type 2 diabetes, as well as the key risk factor obesity, are closely tied to individual social status and regional levels of deprivation [42-44]. However, differences in surveying methods and the quality of data collection also influence incidence and prevalence estimates. In North Rhine-Westphalia, more than 20% of patients were found in general practitioners' practices, a care level that was not comprehensively covered by the survey in Baden-Württemberg. Due to local and historical specificities, there are differences in the diabetological treatment patients receive depending on whether they are living in Baden-Württemberg, Saxony or North Rhine-Westphalia.

### 4.2 Limitations

#### Type 1 diabetes

The analyses only took into account patients with a medical diagnosis of ‘classical’ type 1 diabetes. In routine care, however, without full laboratory analysis, a clear distinction between type 1 diabetes and LADA often cannot be made. Therefore, it cannot be ruled out that a certain number of patients is categorised with the wrong diabetes type. However, the numbers of false categorisations are presumably so small that they do not relevantly influence incidence and prevalence estimates.

The coverage rate for new cases of type 1 diabetes in young adults aged 18 to 34 years was only 30%. The coverage rate for the prevalence of type 1 diabetes in young adults was 73% and, hence, substantially higher. Therefore, a bias in the incidence estimates for North Rhine-Westphalia cannot be entirely ruled out; prevalence estimates are to be considered as more valid.

The coverage rate of the DPV registry for type 1 diabetes could only be estimated for young adults (18- to 34-years-old) using the data of the North Rhine-Westphalian registry. In this case too, the coverage rate for incident cases (around 17%) were far lower than for prevalent cases (around 61%). Moreover, it should be noted that uncertainties in the estimation of coverage rates were not considered when estimating adjusted incidences and prevalences.

For coverage rates at older ages, plausible assumptions on the completeness of capture of the DPV registry had to be made. For incident cases, the estimated coverage rate for young adults (18- to 34-years-old) was applied to older age groups, for prevalent cases a coverage rate of 15% was chosen. An underreporting of adult prevalent cases must already be expected, because prevalent type 1 diabetes cases at the age of e.g. over 26 in 2016, who therefore developed diabetes before 1990, were less likely to be registered due to the late start of registries (DPV mid 1990s, North Rhine-Westphalia registry 2002). Assumptions on the coverage rate of the DPV registry used could lead to distortions of national incidence and prevalence estimates. In terms of a sensitivity analysis, however, the assumptions regarding the completeness of capture were varied.

#### Type 2 diabetes

The registries only include diagnosed cases of type 2 diabetes in children and adolescents. They therefore estimate only the incidence and prevalence of known type 2 diabetes. However, a significant number of undiagnosed cases cannot be ruled out, though precise estimates for children and adolescents do not exist. For adulthood, a proportion of unknown type 2 diabetes in the total prevalence of 20% to 50% has been reported [45]. Moreover, a clear distinction between type 2 diabetes and monogenetic diabetes forms is not always easy in children and adolescents, which may lead to erroneous categorisation.

The coverage rate of the North Rhine-Westphalian registry for the incidence and prevalence of type 2 diabetes in 11- to 18-year-old children and adolescents was estimated at 72% or 77%. The corresponding rates in the DPV data for North Rhine-Westphalia were 52% or 59%. The estimates of national incidence and prevalence are therefore more uncertain than the estimates from North Rhine-Westphalia.

For the surveys in Saxony and Baden-Württemberg no formal estimates of total coverage rate were possible. The figures in Saxony were adjusted based on the survey response rate and in Baden-Württemberg based on an earlier estimate of the coverage rate [14]. Due to the anonymised data collection, double reporting could lead to an overestimation if the patient is treated simultaneously in a specialised diabetological practice and an outpatient clinic or if patients move. However, the determined case numbers probably underestimate the true prevalences. The estimates presented here are therefore only rough approximations.

### 4.3 Conclusion and outlook

Initially, the project ‘Type 1 diabetes in adults and type 2 diabetes in children and adolescents’ focused on developing and providing methods and procedures to estimate the incidence and prevalence of type 1 diabetes at adult age and type 2 diabetes at child and adolescent age in close co-operation with the available local registries in Baden-Württemberg, North Rhine-Westphalia and Saxony, as well as the national DPV registry. For paediatric clinics (participants in the diabetes registries in Saxony and Baden-Württemberg) and diabetological clinics, surveys were established on treated child and adolescent type 2 diabetes in Baden-Württemberg (prevalence) and newly diagnosed child and adolescent type 2 diabetes in Saxony (incidence). Furthermore, in a co-operation between the DPV registry Ulm and the diabetes registry in North Rhine-Westphalia, methods and procedures to estimate the national incidence and prevalence of type 1 diabetes in adults and type 2 diabetes in children and adolescents were provided. Gender-specific aspects were also taken into account.

The structures to estimate the national incidence and prevalence of type 1 diabetes in adults and type 2 diabetes in children and adolescents established by this project provide the basis for a continuous surveillance of these epidemiological parameters in the future and therefore also to assess trends over time. The provided data complements the available epidemiological data on child and adolescent type 1 diabetes (three incidence registries in Baden-Württemberg, North Rhine-Westphalia and Saxony, as well as the national DPV registry) and on type 2 diabetes in adults (regional and national surveys, as well as analyses of the data provided by statutory health insurances), so that diabetes surveillance can monitor the epidemiology of type 1 and type 2 diabetes across all age groups. For health policy and public health institutions this is key to planning future health care needs, and is also important for the interested public. Annually updating this data is furthermore important to assess whether prevention measures are effective at the population level.

To consolidate the diabetes surveillance, data on type 1 diabetes in adults and type 2 diabetes in children and adolescents should also be collected and evaluated in the coming years. For the manifestation year 2018 this has already occurred (co-operation project 2019). A further perspective for the future of diabetes surveillance could be to expand the scope beyond type 1 and type 2 diabetes and include analyses of rare forms at national level which are nevertheless relevant for an overall evaluation of diabetes, such as genetic-based (for example MODY diabetes) or secondary diabetes forms (for example in mucoviscidosis patients) because valid epidemiological data on rare forms of diabetes cannot be collected through population-based representative samples [46].

## Key statements

- Currently, around 4,150 adults and 3,100 children and adolescents (aged 0 to 17) develop type 1 diabetes annually.
- Around 341,000 adults and 32,000 children and adolescents have type 1 diabetes.
- Currently, about 175 children and adolescents aged 11 to 18 years develop type 2 diabetes annually.
- Around 950 children and adolescents aged 11 to 18 years have type 2 diabetes.
- Incidence and prevalence of type 1 diabetes is lower in women than in men.

## Figures and Tables

**Table 1:** Indicators and data sources Own table

Indicators	Data sources
Incidence and prevalence of type 1 diabetes in adults	► Diabetes registry of North Rhine-Westphalia ► DPV registry
Incidence and prevalence of type 2 diabetes in children and adolescents	► Diabetes registry of North Rhine-Westphalia ► DPV registry ► Interview survey Saxony (only incidence) ► Interview survey Baden-Württemberg (only prevalence)

DPV = Diabetes patient documentation

**Table 2:** Incidence and prevalence of type 1 diabetes in Germany Source: Diabetes registry of North Rhine-Westphalia, Diabetes patient documentation (DPV registry) [1, 19]

Study population					2014-2016
	Incident cases			Incidence (95% CI)^1^	
	Registered	CR^2^	Adjusted^3,4^	Registered^5^	Adjusted^3^
**North Rhine-Westphalia 18-34 years**					
Total	391	29.5%	**1,326**	3.6 (3.2-3.9)	**12.0 (11.4-12.7)**
Women	138	32.6%	**423**	2.6 (2.2-3.1)	**7.9 (7.2-8.7)**
Men	253	27.8%	**911**	4.5 (3.9-5.1)	**16.1 (15.1-17.2)**
18-24 years	184	32.3%	**569**	4.2 (3.6-4.8)	**12.9 (11.9-14.0)**
25-34 years	207	26.8%	**772**	3.2 (2.7-3.6)	**11.7 (10.9-12.5)**
**Germany ≥18 years**					
Total	2,144	17.2%^6^	**12,495**	1.0 (1.0-1.1)	**6.1 (6.0-6.2)**
Women	895	15.7%^6^	**5,685**	0.9 (0.8-0.9)	**5.4 (5.3-5.5)**
Men	1,249	21.3%^6^	**5,857**	1.2 (1.2-1.3)	**5.8 (5.7-6.0)**
18-24 years	436	17.2%	**2,541**	2.3 (2.1-2.5)	**13.5 (12.9-14.0)**
25-34 years	546	17.2%	**3,182**	1.7 (1.6-1.9)	**10.1 (9.8-10.5)**
35-44 years	367	17.2%^7^	**2,139**	1.2 (1.1-1.4)	**7.2 (6.9-7.5)**
45-54 years	384	17.2%^7^	**2,238**	1.0 (0.9-1.1)	**5.6 (5.3-5.8)**
55-64 years	226	17.2%^7^	**1,317**	0.7 (0.6-0.8)	**3.9 (3.7-4.1)**
65-74 years	122	17.2%^7^	**711**	0.5 (0.4-0.6)	**2.9 (2.6-3.1)**
≥75 years	63	17.2%^7^	**367**	0.2 (0.2-0.3)	**1.4 (1.2-1.5)**

CR = coverage rate, ESPED = German Paediatric Surveillance Unit, DPV = diabetes patient documentation, AICC = Akaike information criterion, adjusted version for small samples, CI = Confidence interval

^1^ per 100,000 person-years

^2^ Estimated coverage rate based on capture-recapture analysis using three sources of data (ESPED, practices, DPV) according to best log linear model based on AICC criterion with main effects ESPED, practices and DPV for models of incidence and prevalence, and interactions ESPED*practices and ESPED*DPV for prevalence models for the data from the diabetes registry North Rhine-Westphalia (type 1 diabetes 18- to 34-years-old, type 2 diabetes 11- to 18-years-old)

^3^ Adjusted according to the estimated underreporting

^4^ The sum of adjusted case numbers for both genders and/or age groups may not correspond to the adjusted case number for the total group because the number of cases is estimated based on different log linear models

^5^ Estimated based on registered number of cases

^6^ Estimated as sum of all captured cases across all age groups divided by the sum of all cases adjusted by under-coverage for all age groups

^7^ Assumed DPV coverage rate according to the 18- to 34-year-old age group

**Table 2 Continued:** Incidence and prevalence of type 1 diabetes in Germany Source: Diabetes registry of North Rhine-Westphalia, Diabetes patient documentation (DPV registry) [1, 19]

Study population					2016 (2015, 2014)	
	Prevalent cases			Prevalence (95% CI)^1^		
	Registered	CR^2^	Adjusted^3,4^	Registered^5^	Adjusted^3^	
**North Rhine-Westphalia 18-34 years**						
Total	11,284	72.5%	**15,554**	303.8 (298.3-309.5)	**418.8 (412.3-425.4)**	
	10,803	70.4%	**15,352**	290.9 (285.4-296.4)	**413.4 (406.9-420.0)**	(2015)
	10,291	67.2%	**15,313**	286.6 (281.1-292.2)	**426.4 (419.7-433.2)**	(2014)
Women	5,123	72.9%	**7,030**	285.7 (277.9-293.6)	**392.1 (382.9-401.3)**	
Men	6,161	72.5%	**8,499**	320.8 (312.8-328.9)	**442.5 (433.1-452.0)**	
18-24 years	5,099	88.6%	**5,754**	344.1 (334.7-353.7)	**388.3 (378.4-398.5)**	
25-34 years	6,185	59.6%	**10,379**	277.1 (270.2-284.1)	**465.0 (456.1-474.0)**	
**Germany ≥18 years**						
Total	83,215	24.4%^6^	**340,664**	120.5 (119.7-121.3)	**493.3 (491.7-495.0)**	
	79,812	24.1%^6^	**331,203**	115.9 (115.1-116.7)	**481.1 (479.4-482.7)**	(2015)
	76,450	23.6%^6^	**324,393**	112.3 (111.5-113.1)	**476.4 (474.8-478.1)**	(2014)
Women	38,733	24.7%^6^	**157,063**	109.7 (108.6-110.8)	**445.0 (442.8-447.2)**	
Men	44,582	24.2%^6^	**183,446**	131.8 (130.6-133.0)	**543.5 (541.0-546.0)**	
18-24 years	21,259	79.1%	**26,879**	336.1 (331.6-340.6)	**425.0 (419.9-430.0)**	
25-34 years	22,978	42.6%	**53,932**	217.0 (214.2-218.4)	**509.4 (505.1-513.7)**	
35-44 years	10,131	15.0%^7^	**67,540**	102.5 (100.5-104.5)	**683.6 (678.4-688.8)**	
45-54 years	9,702	15.0%^7^	**64,680**	73.3 (71.8-74.7)	**488.4 (484.6-492.1)**	
55-64 years	8,072	15.0%^7^	**53,813**	70.2 (68.6-71.7)	**467.8 (463.8-471.7)**	
65-74 years	5,229	15.0%^7^	**34,860**	63.6 (61.9-65.4)	**424.2 (419.7-428.6)**	
≥75 years	5,844	15.0%^7^	**38,960**	62.9 (61.3-64.5)	**419.3 (415.3-423.5)**	

CR = coverage rate, ESPED = German Paediatric Surveillance Unit, DPV = diabetes patient documentation, AICC = Akaike information criterion, adjusted version for small samples, CI = Confidence interval

^1^ per 100,000 persons

^2^ Estimated coverage rate based on capture-recapture analysis using three sources of data (ESPED, practices, DPV) according to best log linear model based on AICC criterion with main effects ESPED, practices and DPV for models of incidence and prevalence, and interactions ESPED*practices and ESPED*DPV for prevalence models for the data from the diabetes registry North Rhine-Westphalia (type 1 diabetes 18- to 34-years-old, type 2 diabetes 11- to 18-years-old)

^3^ Adjusted according to the estimated underreporting

^4^ The sum of adjusted case numbers for both genders and/or age groups may not correspond to the adjusted case number for the total group because the number of cases is estimated based on different log linear models

^5^ Estimated based on registered number of cases

^6^ Estimated as sum of all captured cases across all age groups divided by the sum of all cases adjusted by under-coverage for all age groups

^7^ Assumed DPV coverage rate according to the 18- to 34-year-old age group

**Table 3:** Incidence and prevalence of type 2 diabetes in Germany Source: Baden-Württemberg DIARY (Diabetes Incidence Registry), diabetes registry of North Rhine-Westphalia, diabetes registry of Saxony, Diabetes patient documentation (DPV registry) [1, 19-23]

Study population					2014-2016	
	Incident cases			Incidence (95% CI)^1^		
	Registered	CR^2^	Adjusted^3,4^	Registered^5^	Adjusted^3^	
**North Rhine-Westphalia 11-18 years**						
Total	105	71.9%	**146**	2.4 (2.0-3.0)	**3.4 (2.9-4.0)**	
Girls	63	72.4%	**87**	3.0 (2.3-3.9)	**4.2 (3.4-5.2)**	
Boys	42	72.4%	**58**	1.9 (1.4-2.6)	**2.6 (2.0-3.4)**	
18-24 years	53	74.6%	**71**	2.6 (2.0-3.4)	**3.5 (2.7-4.4)**	
25-34 years	52	69.2%	**75**	2.3 (1.7-3.0)	**3.3 (2.6-4.1)**	
**Saxony 11-18 years**						
	10	90.9%^6^	**11**	4.0 (1.5-6.4)	**4.3 (1.8-6.9)**	(2016)
	21	84.0%^6^	25	4.2 (2.4-6.1)	**5.1 (3.1-7.1)**	(2014/2015)^7^
**Germany 11-18 years**						
Total	273	51.6%	**529**	1.5 (1.3-1.6)	**2.8 (2.6-3.1)**	
Girls	172	55.1%	**312**	1.9 (1.6-2.2)	**3.4 (3.1-3.9)**	
Boys	101	45.7%	**221**	1.1 (0.9-1.3)	**2.3 (2.0-2.6)**	
11-14 years	81	57.7%	**140**	0.9 (0.7-1.1)	**1.6 (1.3-1.9)**	
15-18 years	192	49.3%	**389**	2.0 (1.7-2.3)	**4.0 (3.6-4.4)**	

CR = coverage rate, ESPED = German Paediatric Surveillance Unit, DPV = diabetes patient documentation, AICC = Akaike information criterion, adjusted version for small samples, CI = Confidence interval

^1^ per 100,000 person-years

^2^ Estimated coverage rate based on capture-recapture analysis using three sources of data (ESPED, practices, DPV) according to best log linear model based on AICC criterion with main effects ESPED, practices and DPV for models of incidence and prevalence, and interactions ESPED*practices and ESPED*DPV for prevalence models for the data from the diabetes registry North Rhine-Westphalia (type 1 diabetes 18- to 34-years-old, type 2 diabetes 11- to 18-years-old)

^3^ Adjusted according to the estimated underreporting

^4^ The sum of adjusted case numbers for both genders and/or age groups may not correspond to the adjusted case number for the total group because the number of cases is estimated based on different log linear models.

^5^ Estimated based on registered number of cases

^6^ Response rate for surveys of paediatric and diabetological practices in Saxony

^7^ Estimated total coverage rate by [14]

**Table 3 Continued:** Incidence and prevalence of type 2 diabetes in Germany Source: Baden-Württemberg DIARY (Diabetes Incidence Registry), diabetes registry of North Rhine-Westphalia, diabetes registry of Saxony, Diabetes patient documentation (DPV registry) [1, 19-23]

Study population					2016 (2015, 2014)	
	Prevalent cases			Prevalence (95% CI)^1^		
	Registered	CR^2^	Adjusted^3,4^	Registered^5^	Adjusted^3^	
**North Rhine-Westphalia 11-18 years**						
Total	150	77.3%	**194**	10.4 (8.8-12.2)	**13.5 (11.7-15.5)**	
	167	76.6%	**218**	11.6 (9.9-13.5)	**15.2 (13.2-17.3)**	(2015)
	165	78.2%	**211**	11.5 (9.8-13.4)	**14.7 (12.8-16.9)**	(2014)
Girls	91	76.5%	**119**	13.1 (10.6-16.1)	**17.2 (14.2-20.6)**	
Boys	59	78.7%	**75**	7.9 (6.0-10.2)	**10.1 (7.9-12.6)**	
11-14 years	30	71.4%	**42**	4.5 (3.0-6.4)	**6.2 (4.5-8.4)**	
15-18 years	120	78.4%	**153**	15.7 (13.0-18.8)	**20.0 (17.0-23.5)**	
**Baden-Württemberg <20 years**						
	80	90.0%^6^	89	3.8 (3.0-4.6)	**4.2 (3.4-5.2)**	(2016)
	50	90.0%^6^	56	2.4 (1.8-3.1)	**2.7 (2.0-3.5)**	(2015)^6^
**Germany 11-18 years**						
Total	445	58.7%	**758**	7.1 (6.5-7.8)	**12.2 (11.3-13.0)**	
	554	57.2%	**968**	8.8 (8.1-9.5)	**15.4 (14.4-16.3)**	(2015)
	652	57.4%	**1,136**	10.5 (9.7-11.3)	**18.2 (17.2-19.3)**	(2014)
Girls	273	60.4%	**452**	9.1 (8.0-10.2)	**15.1 (13.7-16.5)**	
Boys	172	55.8%	**308**	5.3 (4.5-6.1)	**9.6 (8.5-10.6)**	
11-14 years	106	54.8%	**194**	3.6 (2.9-4.3)	**6.6 (5.6-7.5)**	
15-18 years	339	60.1%	**564**	10.4 (9.3-11.5)	**17.2 (15.8-18.7)**	

CR = coverage rate, ESPED = German Paediatric Surveillance Unit, DPV = diabetes patient documentation, AICC = Akaike information criterion, adjusted version for small samples, CI = Confidence interval

^1^ per 100,000 persons

^2^ Estimated coverage rate based on capture-recapture analysis using three sources of data (ESPED, practices, DPV) according to best log linear model based on AICC criterion with main effects ESPED, practices and DPV for models of incidence and prevalence, and interactions ESPED*practices and ESPED*DPV for prevalence models for the data from the diabetes registry North Rhine-Westphalia (type 1 diabetes 18- to 34-years-old, type 2 diabetes 11- to 18-years-old)

^3^ Adjusted according to the estimated underreporting

^4^ The sum of adjusted case numbers for both genders and/or age groups may not correspond to the adjusted case number for the total group because the number of cases is estimated based on different log linear models.

^5^ Estimated based on registered number of cases

^6^ Estimated total coverage rate by [14]
